# This Is Your Fly's Brain on Drugs

**DOI:** 10.1371/journal.pbio.0020444

**Published:** 2004-11-23

Cocaine addiction wreaks profound changes on the brain, hijacking reward circuits and depressing inhibitory loops to the point that drug seeking and taking become central drivers of behavior. Lying at the core of these behavioral changes are molecular ones; at its most basic level, addiction alters the sensitivity of neurons. While primates and rats are useful for mapping out the neural complexity of these behavioral manifestations, insights into the molecular basis of drug abuse can be garnered more easily from simpler models, such as the fruitfly, Drosophila.

The reigning model of cocaine's effects on the brain has highlighted its ability to block reuptake of dopamine by cells of a brain region called the nucleus accumbens. But numerous experiments show this is not the whole story. Ulrike Heberlein and colleagues describe their discovery of a new gene that modulates sensitivity to cocaine within the cells of the fruitfly's internal clock. They further show that the cells' role in regulating cocaine sensitivity is distinct from its function as a timekeeper.

One known effect of cocaine on Drosophila is loss of “negative geotaxis,” or wall climbing, in response to startle. Using this behavior to screen 400 different mutants, the researchers identified seven with an increased response to cocaine, and for two of these, the disrupted gene was the same, *Lmo*. The *Lmo* protein, whose levels were reduced by the mutations, is known to regulate certain transcription factors during development. Despite this, no developmental defects were detected in the loss-of-function mutants that might explain the cocaine effect. The researchers also found that a third mutation in the same gene, previously associated with disruption in wing formation, *increased* levels of the *Lmo* protein, and *decreased* response to cocaine. Thus, *Lmo* appears to play a central role in regulating cocaine sensitivity.

While *Lmo* is found throughout the body, it is enriched in the brain, and by expressing normal *Lmo* in oversensitive mutants, Heberlein and colleagues discovered that its cocaine-related effects were localized to the ventral lateral neurons (LN_v_s). Comprising about ten cells per hemisphere, these neurons provide the fly with an internal clock, driving circadian activities even in the absence of light. Not surprisingly, *Lmo* mutants had weaker circadian rhythms than normal flies.

But is increased cocaine sensitivity a simple consequence of a broken clock? Apparently not. To date, the only known output of the LN_v_ is a small peptide called PDF, and PDF mutation causes circadian disruptions. It does not, however, alter cocaine sensitivity. Furthermore, completely obliterating the LN_v_, or blocking its ability to fire, disrupted circadian rhythmicity but *reduced* cocaine sensitivity, rather than increasing it. These results indicate than the LN_v_ normally enhances sensitivity to cocaine, a function enhanced further by *Lmo* mutants, and does so independently of circadian regulation.

Based on their results, Heberlein and colleagues propose a possible model for *Lmo'*s role in modulating cocaine sensitivity. Drawing on recent evidence that a subset of LN_v_ cells possess dopamine receptors, they suggest that *Lmo* expression normally regulates the density of these receptors on LN_v_ cells. Loss of *Lmo* would raise the number of receptors, thereby increasing the sensitivity to cocaine. A key prediction from their findings is that the LN_v_ has another output, as yet undetected, in parallel with PDF that mediates responsiveness to cocaine.

Because *Lmo*-related proteins are found in key areas of mammalian brains, these results may have important implications for understanding innate differences in sensitivity to cocaine in humans, and potentially provide targets for development of drugs to treat or prevent addiction.

